# Genetic Biofortification to Enrich Rice and Wheat Grain Iron: From Genes to Product

**DOI:** 10.3389/fpls.2019.00833

**Published:** 2019-07-16

**Authors:** Yvonne Ludwig, Inez H. Slamet-Loedin

**Affiliations:** Trait and Genome Engineering Cluster, Strategic Innovation Platform, International Rice Research Institute, Los Baños, Philippines

**Keywords:** biofortification, iron, rice, wheat, genes

## Abstract

The micronutrient iron (Fe) is not only essential for plant survival and proliferation but also crucial for healthy human growth and development. Rice and wheat are the two leading staples globally; unfortunately, popular rice and wheat cultivars only have a minuscule amount of Fe content and mainly present in the outer bran layers. Unavailability of considerable Fe-rich rice and wheat germplasms limits the potential of conventional breeding to develop this micronutrient trait in both staples. Agronomic biofortification, defined as soil and foliar fertilizer application, has potential but remains quite challenging to improve grain Fe to the significant level. In contrast, recent accomplishments in genetic biofortification can help to develop Fe-enriched cereal grains to sustainably address the problem of “hidden hunger” when the roadmap from proof of concept to product and adoption can be achieved. Here, we highlight the different genetic biofortification strategies for rice and wheat and path to develop a product.

## Introduction

“Hidden hunger,” the cause of inadequate intake of key micronutrients, is a major problem globally affecting around 2 billion people worldwide, and 30–40% (GBD 2015 Disease and Injury Incidence and Prevalence Collaborators, 2016) of it is caused by iron (Fe) deficiency anemia (IDA). The people most vulnerable to IDA are women and children. IDA can hamper cognitive and physical development, reduce immunity, and enhance the risk of maternal and perinatal mortality. The breeding target to fulfill the 30% estimated average requirement (EAR) of woman and children recommended by the HarvestPlus program for Fe is 13 μg/g in polished rice or around five to sixfold increase of grain Fe in popular rice. While in wheat, it is 59 μg/g (dry weight) of Fe or around twofold (Bouis et al., 2011).

In developing and less developed countries, it is challenging to provide access to a more diverse diet that can ameliorate the micronutrient deficiency. Biofortification, the enhancement of bioavailable micronutrient in the edible parts of staple food by either conventional plant breeding, biotechnology techniques, or agronomic approaches can help to alleviate malnutrition in the regions where the main source of calories and micronutrients come from staples (Bouis and Saltzman, 2017).

## Plant Fe Uptake and Translocation

Iron is an essential micronutrient in plants and is required frequently in various processes such as photosynthesis, respiration, or chlorophyll biosynthesis. Different strategies are known for the uptake of low soluble Fe(III) oxyhydrate from the rhizosphere in higher plants: (a) Strategy I (non-Graminaceae) implicating reduction of ferric Fe(III) chelate reduction at the root surface to allow absorption of ferrous Fe(II) at plasma membrane, (b) Strategy II (Graminaceae) is the chelation strategy involving mugineic acid (MA) biosynthesis and secretion, and (c) a combination of both (Connorton et al., 2017a).

Major key genes of uptake in Strategy I for dicots and non-grass species were identified from Arabidopsis, namely ferric-chelate reductase oxidase 2 (FRO2) (Robinson et al., 1999), Fe-regulated transporter 1 (IRT1) (Eide et al., 1996), and a large number of H^+^-ATPase (HA) genes that are responsible for proton and phenolic compound excretion into the rhizosphere to enhance the solubility of ferric ions (Kobayashi and Nishizawa, 2012).

**FIGURE 1:**
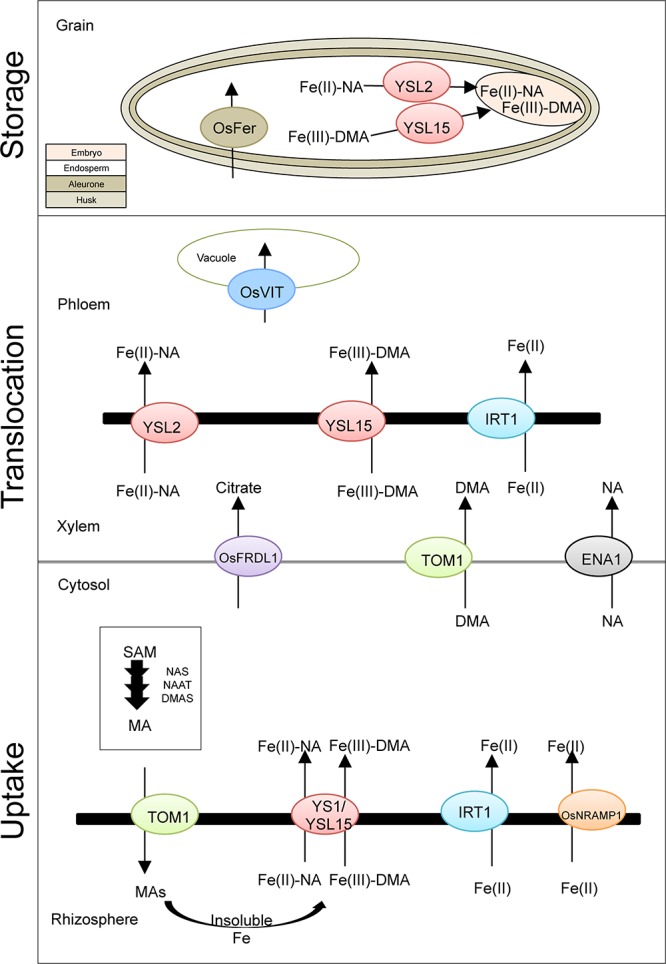
Basic scheme of iron uptake, translocation, and storage in rice. Presented are known genes involved in iron uptake and transport within the rice plant. TOM1, transporter of mugineic acid family phytosiderophores 1; YS1, yellow strip1; YSL15, yellow stripe 1-like; IRT1, iron-regulated transporter; OsNRAMP1, natural resistance-associated macrophage protein 1; OsFRDL1, ferric reductase defective like1; ENA, efflux transporter of nicotianamine; OsVIT, vacuolar iron transporter; OsFER, ferritin; MA, mugineic acid; DMA, 2′-deoxymugineic acid; NA, nicotianamine; SAM, *S*-adenosyl-L-methionine; NAS, nicotianamine synthase; NAAT, nicotianamine aminotransferase; DMAS, deoxymugineic acid synthase.

Strategy II plants such as rice, wheat, barley, and maize secrete the high Fe affinity organic molecule phytosiderophore (PS), a MA family into the rhizosphere (Kobayashi et al., 2005; Suzuki et al., 2006; Borrill et al., 2014; Bashir et al., 2017). The basic scheme for genes involved in Fe homeostasis in rice as the model system is presented in Figure 1. The uptake and translocation process in wheat is similar to rice and has been comprehensively reviewed (Borrill et al., 2014; Connorton et al., 2017a).

The synthesis of MA is a conserved pathway starting from *S*-adenosyl-L-methionine and covers consecutive enzymatic reactions of nicotianamine synthase (NAS), nicotianamine aminotransferase (NAAT), and deoxymugineic acid synthase (DMAS) genes, producing the precursor of nine known types of MAs: 2′-deoxymugineic acid (DMA) (Higuchi et al., 1999; Takahashi et al., 1999; Bashir et al., 2006; Kobayashi and Nishizawa, 2012). In rice, the expression of these genes is highly affected by the level of Fe availability in the soil; most genes were identified in response to Fe deficiency. NAS is localized on the membrane of vesicles in root cells, while NAAT is within these vesicles, proposing the location of the MA biosynthesis. Interestingly, a diurnal pattern is known for the secretion of MA, with its peak in the morning hours (Takagi et al., 1984; Suzuki et al., 2006). The secretion of MA into the rhizosphere is facilitated by the expression of TOM1 (transporter of MA family PSs). TOM1 was first identified in rice and barley and belongs to the major facilitator superfamily (MFS), which is one of the largest group of membrane transport proteins that promote the transit of substrates across cell membranes in response to chemiosmotic ion gradient (Nozoye et al., 2011). Fe–MA complexes are then built and taken up by YELLOW STRIPE 1 (YS1) and YELLOW STRIPE 1-like (YSL1) transporter into root cells (Curie et al., 2001; Inoue et al., 2009). The Fe(III)–DMA complex absorbed in root cell cytosols is likely reduced by ascorbate and altered to be Fe(II)–NA. The cytosolic Fe(II)–NA then excreted to the xylem and created complexes predominantly with citrate or with DMA (Fe–DMA) and transport further (Yoneyama et al., 2015).

In addition to the strategy II Fe(III)–DMA complex and OsYSL15 transporter, rice carries a ferrous transporter (OsIRT1) in its genome, which allows the direct uptake of Fe(II). On the root surface, rice shows a low ferric-chelate reductase activity, indicating an adaptation to submerge and anaerobic conditions (Ishimaru et al., 2006). Other metal transporters were identified for both strategies like ZIP (zinc-regulated transporter, IRT-like protein) family or natural resistance-associated macrophage protein (NRAMP) (Guerinot, 2000; Lanquar et al., 2005; Calliatte et al., 2010).

A complex cascade involves in the Fe translocation in higher plants, including xylem and phloem loading/unloading, transport, and retranslocation within the plant from source to sink organ (Kim and Guerinot, 2007). To facilitate this translocation, different chelators such as citrate, MAs, and nicotianamine (NA) play a crucial role in symplast heavy metal homeostasis (Garcia-Oliveira et al., 2018). The cytosolic Fe(II)–NA then excreted to the xylem and created complexes predominantly with citrate or with DMA (Fe–DMA) and transport further in rice phloem while remaining to bind to DMA, citrate, and proteins (Yoneyama et al., 2015). FERRIC REDUCTASE DEFECTIVE LIKE 1 (OsFRDL1) in rice (Figure 1) encodes a citrate efflux transporter required for Fe translocation. This gene is primarily expressed in root pericycle cells. Knockout plants of OsFRDL1 display a mild defect in Fe homeostasis, which suggests alternative chelators for Fe xylem transport (Inoue et al., 2004; Yokosho et al., 2009).

The YSL family members (influx transporter) are involved in metal–NA chelate translocation (Curie et al., 2009). The rice YSL family consists of 18 members. The OsYSL2 transporter is responsible for the long-distance transfer into sink tissues like leaves and grains (Koike et al., 2004). It is a carrier of Fe(II)–NA, but not Fe(III)–MAs. The transport of Fe(III)–DMA is performed by OsYSL15, which is responsible for Fe root absorption and internal translocation for long distance as well as seedling growth. The Fe transporter OsYSL18 is specifically expressed in reproductive organ, such as the pollen and pollen tube, which suggests a specific role in fertilization. It also can be found in the phloem of laminar joints, indicating a part in phloem Fe transport (Kobayashi and Nishizawa, 2012). The ferrous transporter OsIRT1 and DMA effluxer TOM1 seem not only to be involved in Fe uptake but also in Fe translocation within the plant, due to its expression in vascular tissue in rice (Ishimaru et al., 2006; Nozoye et al., 2011). While the two efflux transporter of NA 1 (ENA1) and ENA2 were involved in NA transport (Nozoye et al., 2011).

## Biofortification: Agronomic Vs./and Genetic in Rice and Wheat

### Agronomic Biofortification

Cereal grain micronutrient content can be enriched through agronomic biofortification, which is a fertilizer-based application method to soil or to plant foliar (Cakmak and Kutman, 2017). Soil agronomic biofortification is easy and can be quite cost-effective (Garg et al., 2018). It is a short time solution, important to complement the genetic biofortification, particularly when the soil in the target region is limited to a readily available pool of micronutrient (Cakmak and Kutman, 2017). For rice, the main challenge is the translocation of the mineral from the vegetative part to the grain (Mabesa et al., 2013; Slamet-Loedin et al., 2015), since it is mostly grown in lowland irrigated areas where Fe is highly available.

Foliar feeding is established via plant tissue test or visual foliar. It is affected by endogenous (leaf anatomy), exogenous (pH, soil), as well as environmental factors. After foliar Fe application, it takes the plants 10–20 days to absorb 50% of the micronutrient (Alshall and El-Ramady, 2017). In contrast with the promising results of foliar fertilizer application to improve grain Zn in rice (Cakmak, 2008; Cakmak and Kutman, 2017), the increase of grain Fe using foliar fortification was modest. A similar phenomenon was observed in wheat, neither soil nor foliar applications in inorganic form (e.g., FeSO_4_) or chelated form (e.g., Fe–EDTA, Fe–EDDHA, or Fe–citrate) were reported to be effective for increasing grain Fe concentrations (Cakmak, 2008). A minor increase in grain Fe was observed with Fe-EDTA application and also nitrogen application in wheat (Cakmak and Kutman, 2017). Another study showed a foliar application of Fe–amino acid (Fe AA) modestly increased grain Fe concentration by 14.5% on average in rice and by 32.5% when 1% (w/v) NA was added (Yuan et al., 2012). A foliar application to reach a significant increase in grain Fe for biofortification remains challenging.

### Genetic Biofortification

Efforts to enrich Fe in the rice grain by conventional breeding are constrained by the limited natural variation of polished grain Fe in rice germplasm. Over 20,000 different accessions of rice germplasm were tested and displayed a maximum of only 5–8 mg kg^–1^ in polished rice grains (Gregorio et al., 2000; Graham, 2003). To date none of the conventional breeding and molecular marker approaches reached grain Fe breeding target in rice and wheat to fulfill 30% EAR in women and children; therefore, this review focuses on the genetic biofortification through transgenic approaches.

To facilitate an efficient and targeted genetic biofortification in rice five key steps can be addressed: (a) enhanced uptake, (b) increase translocation to grain, (c) specialization of Fe storage toward endosperm, (d) decrease of anti-nutritions, and (e) increase of bioavailability (Mulualem, 2015). Both single approach or combination of multiple approaches have been applied in genetic biofortification. The first attempt to obtain an Fe-enriched rice was reported by Goto et al. (1999, Table 1), focusing on improving the Fe storage protein. An increase of grain Fe concentration was attained in brown rice (up to 38 μg/g) compared to the wildtype Japonica cv. Kitaake (∼14.3 μg/g) through ectopic overexpression (OE) of SoyFerH1 in the endosperm. Several studies followed using a similar approach of OE lines of SoyferH1, differing in the genetic background (Swarna, IR68144, BR29, IR64, M12) and usage of promoters (OsGluB1, OsGtbl, CluB1, GluB4, OsG1b) (Slamet-Loedin et al., 2015). In two attempts, an elevated Fe level in the polished rice (up to ∼9.2 or ∼7.6 μg/g) was obtained and stable over several generations (Khalekuzzaman et al., 2006; Oliva et al., 2014); however, in particular case, there was no significant increase observed (Drakakaki et al., 2000). Not only was the soybean storage gene utilized to improve the Fe levels within the grain, but also the effect of overexpressing the rice ferritin gene (OsFer2) was analyzed. Higher Fe concentrations were found in T3 rice seeds (up to ∼15.9 μg/g) compared to the control Indica cv. Pusa-Sugandh II with ∼7 μg/g (Paul et al., 2012). Other studies focus on the genes for Fe uptake and translocation in the plant such as the development of OE lines of OsYSL15 (Lee et al., 2009a), responsible for the uptake of Fe(III)–DMA, and OsYSL2 (Ishimaru et al., 2010) for the uptake of Fe(II)–NA from the rhizosphere. The OsYSL2 OE study indicated a higher Fe content of ∼7.5 μg/g in polished rice compared to its counterpart (∼1.8 μg/g) in the T1 generation (Table 1). Only minimal elevated Fe concentrations were detected for OsYSL15 OE lines in T1 brown rice (Lee et al., 2009a), similar to the OE of OsYSL9 (Senoura et al., 2017). The alternative approach is to increase the expression of the NAS genes in rice, either by adding a 35S promoter enhancer in OsNAS3 and OsNAS2 gene through T-DNA activation tagging (Lee et al., 2009b, 2012), or by an endosperm OE of OsNAS1 (Zheng et al., 2010), or constitutive OE of OsNAS2 gene (Johnson et al., 2011). The endosperm OE of OsNAS1 resulted in 19 μg/g Fe level in brown rice compared to its wildtype (12 μ/g) and reduced to 5 mg/g after polishing (Zheng et al., 2010). Better results were achieved in the OE OsNAS2 plants with a Fe level-up to 19 μg/g in polished rice (Johnson et al., 2011) in comparison with the Nipponbare wildtype (4.5 μg/g), or in the OsNAS3 and OsNAS2 activation tag plants with 12 and 10 μg/g Fe in milled rice respectively compared to the wildtype (4 μg/g) (Lee et al., 2009b, 2012). A high Fe increase up to 55 μg/g was reported in the endosperm of japonica rice by OE of OsNAS1 and HvNAAT genes (Diaz-Benito et al., 2018). The Fe level is exceptionally high for the starchy endosperm suggesting either Fe contamination or the presence aleurone layer. The Fe level in the earlier generation was 18 μg/g (Banakar et al., 2017b) that already fulfill the nutritive target.

**TABLE 1:** Review on transgenic approaches to develop iron-rich rice and wheat grains.

	**Iron ([c] in μg/g)**		**Generation**	**Seed status**	**Growth condition**	**Cultivar**	**References**
	**TG**	**WT**					
**RICE**	**Genes involved in iron uptake and translocation**						
**Rice gene overexpression**							
OsIRT1	∼12	∼10	T3	Brown	Paddy field	Japonica cv. Dongjin	Lee and An, 2009
TOM1	∼18	∼15	T1	Brown	Hydroponic	Japonica cv. Tsukinohikari	Nozoye et al., 2011
OsYSL15	∼14	∼12	T1	Brown	Paddy field	Japonica cv. Dongjin	Lee et al., 2009a
OsNAS1	up to ∼19	∼12		Brown	Field	Japonica cv. Xiushui 110	Zheng et al., 2010
OsNAS2	∼10	∼4		Milled	Greenhouse	Japonica cv. Dongjin	Lee et al., 2012
OsNAS3	∼12	∼4	T1	Milled	Greenhouse	*Oryza sativa* L. (cv EYI 105)	Lee et al., 2009b
OsYSL2	∼7.5	∼1.8	T1	Polished	Glasshouse	Japonica cv. Tsukinohikari	Ishimaru et al., 2010
OsNAS1, OsNAS2, OSNAS3	up to ∼19	∼4.5	T1	Polished	Glasshouse	Japonica cv. Nipponbare	Johnson et al., 2011
OsNAS1	up to ∼40	∼20	T4	Endosperm	Greenhouse	*Oryza sativa* L. (cv EYI 105)	Diaz-Benito et al., 2018
OsYSL13	∼15	∼11		Brown	Greenhouse	Japonica cv. Zhonghua 11	Zhang et al., 2018
**Overexpression of gene of different species**							
HvNAS1	∼8.5	∼4	T2	Polished	Greenhouse	Japonica cv. Tsukinohikari	Masuda et al., 2009
HvYS1	up to ∼9	∼4	T2	Polished		*Oryza sativa* L. (cv EYI 105)	Banakar et al., 2017a
AtIRT1	up to ∼4.86	∼2.28	T3	Polished	Greenhouse	Japonica cv. Taipei 309	Boonyaves et al., 2016
**Genes involved in storage**							
**Rice gene overexpression**							
OsFer2	up to ∼15.9	∼7	T3	Milled	Greenhouse	Indica cv. Pusa-Sugandh II	Paul et al., 2012
**Soybean gene overexpression**							
SoyferH1	up to ∼38	∼14.3	T1	Brown	Greenhouse	Japonica cv. Kitaake	Goto et al., 1999
SoyferH1	up to ∼25	∼17	T3–T6	Brown	Greenhouse	Japonica cv. Kitaake	Qu et al., 2005
SoyferH1	∼18	∼18	T2	Brown	Greenhouse	Indica cv. M12	Drakakaki et al., 2000
SoyferH1	up to ∼37	∼10	T2	Milled	Screenhouse	Indica cv. IR68144	Vasconcelos et al., 2003
SoyferH1	up to ∼16	∼6.75	BC2F5	Milled	Greenhouse	Indica cv. Swarna	Paul et al., 2014
SoyferH1	up to ∼9.2	∼3.8	T3	Polished	Greenhouse	Indica cv. BR29	Khalekuzzaman et al., 2006
SoyferH1	up to ∼7.6	∼3.3	T4	Polished	Greenhouse	Indica cv. IR64	Oliva et al., 2014
**Genes involved in iron deficiency response**							
**Rice gene overexpression**							
OsIRO2	up to ∼15.5	∼6	T1	Brown	Greenhouse	Japonica cv. Tsukinohikari	Ogo et al., 2011
**Genes involved in inter-cellular/intra-cellular transport and storage**							
**Rice gene silencing/knock-down mutant**							
OsVIT1	∼26	∼20		Brown	Paddy field	Japonica cv. Zhonghua 11	Zhang et al., 2012
OsVIT2	∼28	∼20		Brown	Paddy field	Japonica cv. Dongjin	Zhang et al., 2012
OsVIT2	∼8	∼5		Polished		Japonica cv. Dongjin	Bashir et al., 2013
OsYSL9	up to ∼2.5	1		Polished		Japonica cv. Tsukinohikari	Senoura et al., 2017
OsDMAS1	∼5	∼5		Polished		Japonica cv. Dongjin	Bashir et al., 2017
**Combined strategies**							
**Multigene overexpression**							
PyFerritin+rgMT+phyA	∼22	∼10	T1	Brown	Greenhouse	Japonica cv. Taipei 309	Lucca et al., 2002
OsYSL2+, SoyFerH2+, HvNAS1	up to ∼4	∼1	T3	Polished	Paddy field	Japonica cv. Tsukinohikari	Masuda et al., 2012
HvNAS1, HvNAS1+, HvNAAT, IDS3	up to ∼7.3	∼5.8	T1	Polished	Paddy field	Japonica cv. Tsukinohikari	Suzuki et al., 2008
HvNAS1+, OsYSL2, SoyFerH2+	∼6.3 (∼5.02)	∼3.2 (∼1.46)	T1(T2)	Polished	Greenhouse	Tropical Japonica cv. Paw San Yin	Aung et al., 2013
AtNAS1+, Pvferritin+, Afphytase	up to ∼7	∼1	T1	Polished	Hydroponic	Japonica cv. Taipei 309	Wirth et al., 2009
AtIRT1, PvFERRITIN, AtNAS1	up to ∼10.46	∼2.7	T2	Polished	Greenhouse	Japonica cv. Nipponbare	Boonyaves et al., 2017
GmFERRITIN, OsNAS2	∼15	∼2.5	T3	Polished	Field	Indica cv. IR64	Trijatmiko et al., 2016
AtNAS1, AtFRD3, PvFer	up to ∼11.08	∼2.05	T3	Polished	Greenhouse	Japonica cv. Nipponbare	Wu et al., 2018
AtNAS1, PvFer, AtNRAMP3	up to ∼13.65	∼2.72	T2	Polished	Greenhouse	Indica cv. IR64	Wu et al., 2019
AtNAS1, PvFER, ZmPSY, PaCRT1	up to ∼6.02	∼1.82	T3	Polished	Greenhouse	Japonica cv. Nipponbare	Singh et al., 2017a
OsNAS1, HvNAATb	up to ∼55	∼20	T4	Endosperm	Greenhouse	*Oryza sativa* L. (cv EYI 105)	Diaz-Benito et al., 2018
OsNAS1, HvHAATb	∼up to 18	∼4	T3	Endosperm	Hydroponic	*Oryza sativa* L. (cv EYI 105)	Banakar et al., 2017b
**WHEAT**							
**Genes involved in iron uptake and translocation**							
**Single gene over expression**							
OsNAS2	up to ∼80	40		Whole grain	Field	*Triticum aestivum* (cv Bob White)	Beasley et al., 2019
OsNAS2	up to ∼22	14		Flour	Field	*Triticum aestivum* (cv Bob White)	Beasley et al., 2019
**Genes involved in storage**							
Ta FERRITIN	Up to ∼130	72	T2	Whole grain	Greenhouse	*Triticum aestivum* (cv Bob White)	Borg et al., 2012
Ta FERRITIN	Average 88.5	70	T2	Flour	*Greenhouse*	*Triticum aestivum* (cv Bob White)	Borg et al., 2012
**Genes involved in inter-cellular/intra-cellular transport and storage**							
Ta VIT	Up to 20	10	T1	Flour			Connorton et al., 2017b
**Combined strategy**							
**Multigene overexpression**							
OsNAS2 +/or PvFERRITIN	up to ∼93.1	42.7	T4	Whole grain	Greenhouse	*Triticum aestivum* (cv Bob White)	Singh et al., 2017b
OsNAS2, +/or PvFERRITIN	53.3	21.4		Flour	Greenhouse	*Triticum aestivum* (cv Bob White)	Singh et al., 2017b

A few studies use the silencing approach to reduce the Fe content in rice grain. Zhang et al. (2012) developed vascular Fe transport (OsVIT) silencing lines, aiming to interrupt the transport of Fe into the flag leaves. A comparable approach was used by Bashir et al. (2013), achieving a Fe level of ∼8 μg/g in polished rice. Only one gene involved in the Fe deficiency response was used to increase the Fe concentration till now. The constitutive expression of OsIRO2 resulted in ∼15.5 μg/g Fe in T1 brown rice seeds in comparison with the wildtype (∼6 μg/g) (Ogo et al., 2011).

The most promising results for Fe-enriched rice grains in tropical Indica rice were developed by Trijatmiko et al. (2016) and Wu et al. (2019) by multigene OE. By expressing the endosperm storage gene PvFER, the chelator AtNAS1 gene and an intracellular iron stores AtNRAMP3 in one cassette, the level of 13.65 μg/g iron was reached in the greenhouse condition (Wu et al., 2019). A slightly higher level of Fe of 15 μg/g concentration in polished grain Fe coupled with high Zn was shown in the results of Trijatmiko et al. (2016) in two field trials (Table 1; Trijatmiko et al., 2016). Generally, the field condition resulted in a lower grain Fe compared to the glasshouse setting (Masuda et al., 2012). These reported studies serve as a proof of concept for the potential product of Fe-biofortified rice.

In wheat, using the marker-assisted breeding a modest increase in Fe by 18% was observed in near-isogenic lines of NAM-B1 (Distelfeld et al., 2007). The first transgenic approach in wheat reported a significant increase in grain Fe achieved by the OE of the wheat FERRITIN gene (Borg et al., 2012; Table 1). This OE of TaFer1-A gene increased to 50–85% higher Fe content in wheat grains; however, the TaFer1-A genes presence was not stable over the generations (Borg, personal communication). The recent report (Singh et al., 2017b) showed significant progress that OE of OsNAS2 produced up to 93.1 μg/g of Fe in the wheat grain in the greenhouse, it surpasses the recommended nutritive breeding target. While using VACUOLAR IRON TRANSPORTER (TaVIT), a double of Fe concentration was obtained in flour but not in whole grain (Connorton et al., 2017b). Another recent study OE of OsNAS2 in wheat (Beasley et al., 2019) reported up to 80 μg/g of Fe concentration in wheat grain under field condition.

#### Potential of Genome Editing Approaches

A recent development in the genome editing tool, Clustered Regulatory Interspaced Palindromic Repeats (CRISPR) for precise modification within the genome, gives researchers a possibility for accurate targeting of genes or genomic regions. This technology has been used in rice to improve yield and stress resistance (Jaganathan et al., 2018; Mishra et al., 2018). The potential example to use CRISPR-based approach is to knockdown OsVIT2 to achieve the increase of grain Fe, similar to the published T-DNA insertion silencing of this gene (Bashir et al., 2013) in different rice cultivars. The development of Fe-enriched rice and wheat grains can also benefit from this method by tweaking the expression of genes involved in Fe homeostasis by editing the regulatory element of Fe homeostasis genes.

## Trait Selection and Post-Harvest Effect on Iron and Bioavailability

The accuracy of grain Fe measurement requires consistent preparation, contamination free, and a standardized processing method to reach a consistent milling degree to select the product. The most accurate technique for Fe measurement is ICP-OES (Elzain et al., 2016).

Unpolished brown rice and wheat have a higher concentration and variation of Fe in the aleurone layers, wherein most of the Fe in rice and wheat is stored. Fe in cereal is stored in vacuoles in complex with phytate (Borg et al., 2012), and localized in the aleurone layer and embryo parts. Minimal phytate amount has been detected in the endosperm (Saenchai et al., 2016). Depending on the genotype, the rice bran can have one to five aleurone layers (Del Rosario et al., 1968). Unfortunately, dietary factors that inhibit cereal Fe absorption in human include phytic acid (myoinositol hexaphosphate) together with polyphenols (Gibson et al., 2010; Moretti et al., 2014), tannins, fiber, or hemagglutinins (Welch and Graham, 2004; Holme et al., 2012). Fe in cereal bran is chelated by phytic acid and forms an insoluble complex in the gastrointestinal tract (Iqbal et al., 1994). Rice is predominantly consumed in polished form, while wheat consumption is mixed between brown and white flour. To effectively reduce the “hidden hunger,” it is crucial that the added biofortified grain Fe is bioavailable.

Iron bound to ferritin is most likely to be highly available (Lönnerdal et al., 2006). NA has also been suggested as a Fe bioavailability enhancer in rice (Zheng et al., 2010). The correlation of the bioavailability of grain Fe with the presence of NA and ferritin was shown in rice (Trijatmiko et al., 2016). Recently in wheat, the OE lines of OsNAS2 show that the endosperm Fe is not co-localized with phosphorous but likely with NA (Beasley et al., 2019).

## Pathway of a Genetic Biofortification: Proof of Concept to a Product

The path from proof of concept to the product started from the development of commercial event amenable for deregulation, the trait and agronomic testing phase in multiple confined fields, the regulatory science phase, and varietal registration phase (McDougall, 2011). To pass the regulatory requirement, GM rice needs to be screened for single locus insertion containing one or more copies and characterized at the molecular level of the actual insert, the absence of vector backbone or other unintended change, and heritability and followed by comprehensive food and environmental safety studies to prove product safety (Heck et al., 2005). In the biofortification study by Trijatmiko et al. (2016), the highest concentrations were obtained in the lines having two copies of transgenes in one insertion locus. Insertion of more than one copy of a transgene does not impede event deregulation as has been shown in some commercially released crops events (Canola-23-18-17, Soya-GU262) with multiple copies (Health Canada, 1999; ISAAA, 2019).

Collection of a comprehensive safety data of food and feed use of Fe-biofortified rice to develop a robust safety regulatory dossier is crucial. In most countries, the assessment follows the Codex Alimentarius guideline, which requires the description of the donor organism; genetic modification and its characterization; and assessment of the possible toxicity, allergenicity, and compositional analysis (FAO, 2003). For the environmental evaluation of nutrition trait, the necessary assessment could be to monitor the relevant insect pest population and change in seed vigor to assess the weediness potential.

Development and submission of a robust biosafety dossier, an excellent agronomic performance, enhancement of Fe bioavailability, and bioefficacy (Bouis and Saltzman, 2017) within the local context of the staple food processing, cooking, and eating habit, and a strong support of the seed sector and health sector in target countries will lay a path for deployment and adoption of Fe-enriched rice. Significant progress in genetic biofortification in recent years can provide a sustainable food-based solution to complement other interventions to reduce iron deficiency anemia in the target communities.

## Author Contributions

Both authors listed have made a substantial, direct and intellectual contribution to the work, and approved it for publication.

## Conflict of Interest Statement

The authors declare that the research was conducted in the absence of any commercial or financial relationships that could be construed as a potential conflict of interest.
